# American Orthopedic Association—The Embodiment of an Idea

**Published:** 1893-11

**Authors:** A. J. Steele

**Affiliations:** St. Louis, Mo.


### AMERICAN ORTHOPEDIC ASSOCIATION.—THE EMBODIMENT OF AN IDEA.

PRESIDENTIAL ADDRESS BY DR. A. J. STEELE, ST. LOUIS, MO.

(Abstract.)

The conception of the mind becomes through action a reality. The magnificent St. Louis Bridge is the embodied idea of the engineer, James B. Eads. The foundations are marvels of solidity; the arches of grace and strength. The orthopedic appliances of the present day are the result of thought. Education of both the mind and hand is a prerequisite of good work.

The mission of the orthopedist is to save life, to correct deformities, to restore action, and to change from helplessness to usefulness. No copyrights or patents are found on our appliances. We give the world the benefit of our ideas and experience. Ten years ago the Orthopedic Society was a dream, today a reality. We are a recognized specialty increasing in numbers, and with valuable publications. Our annual printed volume should contain not only our own choicest thoughts, but also an epitome of the progress of orthopedic surgery of the world. We have much of charity work because the mass of our cases are from among the poor.

Not many years ago even the profession inquired the meaning of orthopedics, but now the people are becoming familiar with our work, and come directly to us. It is a noteworthy fact that all our members are engaged in public work, dispensary, hospital, or college. Great is the benefit that accrues to the teacher in obtaining a mastery of his subject. Orthopedics is interesting to the student, for there is so much of object illustration in it, bone specimens, casts, appliances, and the patients and the ever attractive operation. Our mission, the prevention and cure of deformities, will include a thorough knowledge of joint disease, and the performance of any required operation; so we are not merely splint doctors.

The young graduate should not think of stepping at once into a specialty; years of special training and general practice are necessary precursors before limiting one's self to a single department of practice.

As deformity induces moroseness and misanthropy, so as we cure our patients, their dispositions are changed to cheerfulness and their bodies to usefulness.

Among hinderances to our success are, on our own part, want of ability and unfortunate mannerism; outside of ourselves, the natural bonesetter, the traveling quack, the orthopedic institute, the pretentious instrument maker. The day hastens when legislation will drive the charlatan to the wall, and the education of the masses prompt them to at once seek the orthopedist when needed. Orthopedic surgery is yet young, but a great future is before it, in the making of which this Association will ever be a strong factor.

